# Flap Reconstruction for Deep Hand Burns: A Case Series

**DOI:** 10.7759/cureus.116077

**Published:** 2026-09-10

**Authors:** Junya Oshima, Kaoru Sasaki, Mitsuru Sekido

**Affiliations:** 1 Department of Plastic, Reconstructive, and Hand Surgery, University of Tsukuba, Tsukuba, JPN

**Keywords:** abdominal flap, deep hand burn, flap reconstruction, hand reconstruction, heat-press injury

## Abstract

Introduction

Deep hand burns are severe injuries that frequently result in exposure of tendons and bone because of the thin subcutaneous tissue of the hand. In such cases, skin grafting alone is often insufficient, and flap reconstruction is required. Heat-press injuries are particularly challenging because combined thermal and crush mechanisms make tissue viability difficult to evaluate. The optimal timing of debridement and reconstruction also remains controversial.

Methods

We retrospectively reviewed the medical records of six patients (six hands) with acute deep hand burns distal to the wrist who required flap reconstruction between April 2004 and April 2025. Clinical variables including injury mechanism, injury location, burn depth, timing of surgery, reconstructive procedures, and outcomes were evaluated.

Results

The study included three (50.0%) male and three (50.0%) female patients ranging in age from 21 to 61 years. Four (66.7%) injuries were caused by heat-press mechanisms, one (16.7%) by contact with heated metal, and one (16.7%) by flame burn. Three (50.0%) patients had injuries limited to the digits, one (16.7%) patient had injuries extending from the digits to the dorsal aspect of the hand, and two (33.3%) patients had injuries extending from the digits to the palm and dorsum. Five (83.3%) patients had grade 3 injuries according to the Tajima classification. Debridement and flap reconstruction were performed simultaneously within one to 10 days of injury in four (66.7%) patients. In one (16.7%) patient with extensive burns, flap reconstruction was delayed until 64 days after injury because treatment of the life-threatening burns was prioritized. In another patient (16.7%), delayed necrosis became clinically apparent after the initial debridement performed 11 days after injury, requiring additional reconstruction with a free anterolateral thigh flap 42 days after injury. Ultimately, digital amputation was required in three (50.0%) patients. Patients with predominantly dorsal injuries achieved relatively favorable outcomes, whereas palmar or combined palmar-dorsal injuries were associated with poor prognosis.

Conclusions

Deep hand burns involving the palmar aspect or both the palmar and the dorsal aspects carry a high risk of progressive necrosis and amputation despite flap reconstruction. Our findings suggest that early coverage using well-vascularized flaps and carefully staged treatment strategies considering delayed necrosis may be important for tissue preservation and functional reconstruction.

## Introduction

Deep hand burns are severe injuries that directly affect functional outcomes. In particular, deep thermal injuries frequently result in exposure of tendons and bone because the hand has thin subcutaneous tissue and is anatomically vulnerable to deep thermal damage [1]. In such cases, wound closure using skin grafting alone is often insufficient, and flap reconstruction is required.

Heat-press injury, a combined thermal and crush injury, is known to cause progressive tissue damage, making assessment of tissue viability particularly difficult. In addition, the optimal timing of debridement and flap coverage remains unclear.

The aim of this study was to describe the clinical course and outcomes of deep hand burns requiring flap reconstruction, with particular attention to injury pattern, timing of reconstruction, and digital salvage.

## Materials and methods

We retrospectively reviewed the medical records of six patients (six hands) with acute hand burns distal to the wrist who required flap reconstruction and were treated at our institution and affiliated hospitals between April 2004 and April 2025.

The following variables were evaluated: sex, age, affected side, mechanism of injury, injury location, depth of injury, timing of surgery, reconstructive procedure, follow-up period, clinical outcome, and need for secondary reconstruction.

Injury locations were classified into the digits, palm, and dorsum of the hand. Burn depth was evaluated according to the heat-press injury classification proposed by Tajima [2]. According to this classification, grade 1 injuries were defined as tissue necrosis extending from the full thickness of the skin to the surface of the deep fascia or tendon. Grade 2 injuries were defined as necrosis involving the deep fascia or extensor tendon (dorsal aponeurosis in the digits) while sparing the periosteum and bone. Grade 3 injuries were defined as injuries involving partial necrosis of the bone. Multiple Tajima grades could coexist within the same hand according to the depth of injury at each site.

## Results

The study included three (50.0%) male and three (50.0%) female patients ranging in age from 21 to 61 years. All six (100%) injuries involved the right hand. The mechanisms of injury included heat-press injury in four (66.7%) patients, contact with heated metal in one (16.7%) patient, and flame burn in one (16.7%) patient. The follow-up period ranged from three months to four years after injury.

Regarding the injury location, three (50.0%) patients had injuries limited to the digits, one (16.7%) patient had injuries extending from the digits to the dorsal aspect of the hand, and two (33.3%) patients had injuries extending from the digits to the palm and dorsum. Five (83.3%) patients had injuries including grade 3 burns according to the Tajima classification.

In four (66.7%) patients, debridement and flap reconstruction were performed simultaneously within one to 10 days after injury. In one (16.7%) patient with extensive burns, flap reconstruction was delayed until 64 days after injury because treatment of the life-threatening burns was prioritized. In another one (16.7%) patient, delayed necrosis became clinically apparent after the initial debridement performed 11 days after injury, requiring additional reconstruction with a free anterolateral thigh flap 42 days after injury.

Ultimately, digital amputation was required in three (50.0%) patients. Secondary reconstruction procedures included thumb reconstruction with toe transfer in one (16.7%) patient and extensor tendon reconstruction in another one (16.7%) patient (Figure 1, Table 1).

**Figure 1:**
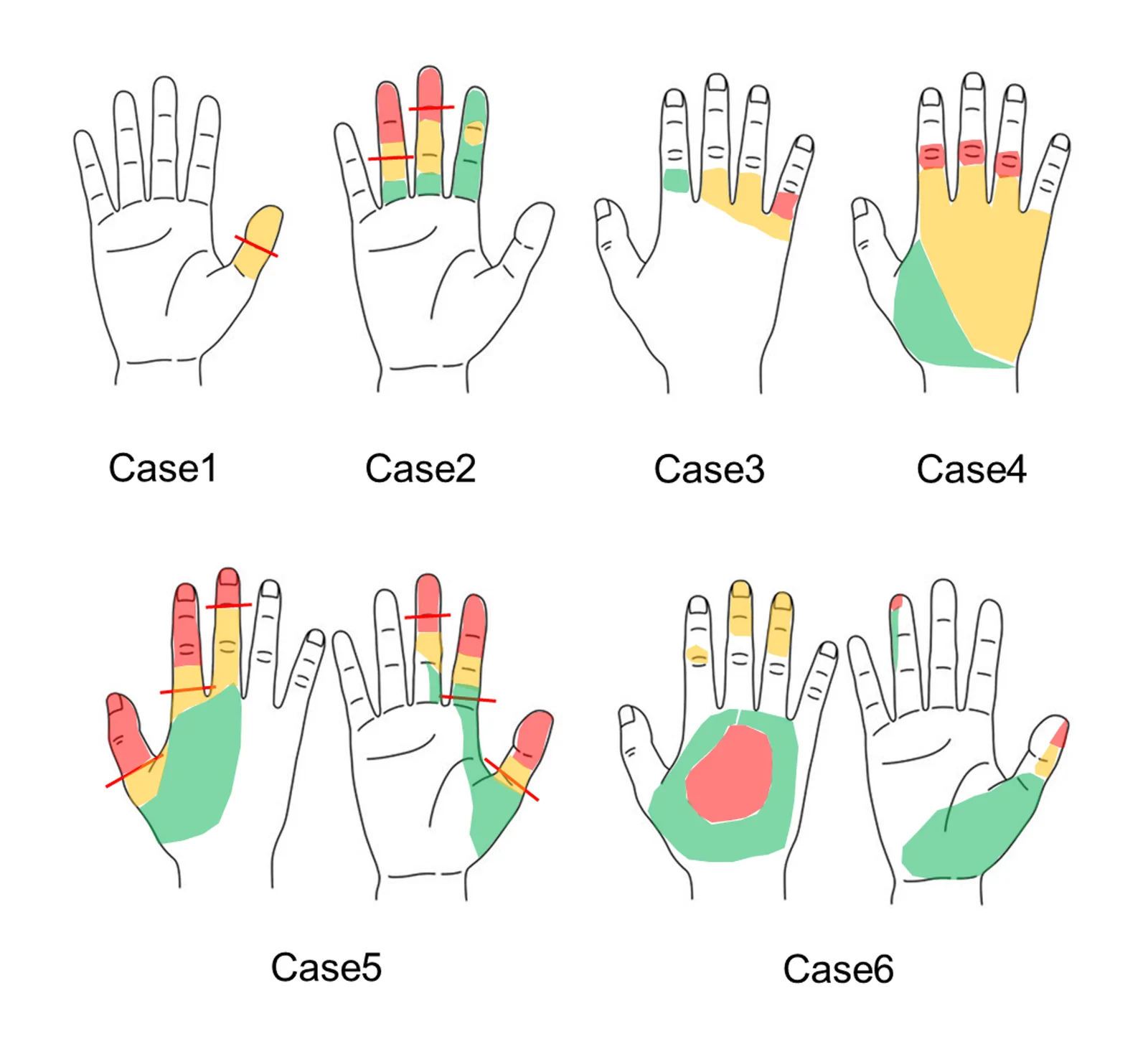
Schematic illustration of the extent and depth of the burns Burn depth was classified according to the Tajima classification: grade 1 injuries (green) indicate tissue necrosis extending from the full thickness of the skin to the surface of the deep fascia or tendon; grade 2 injuries (yellow) indicate necrosis involving the deep fascia or extensor tendon while sparing the periosteum and bone; grade 3 injuries (red) indicate partial necrosis of the bone. The red lines indicate the level of amputation performed during the clinical course.

**Table 1:** Clinical characteristics and outcomes of six patients with deep hand burns requiring flap reconstruction

Characteristic	Case 1	Case 2	Case 3	Case 4	Case 5	Case 6
Sex	Male	Male	Male	Female	Female	Female
Age, year	32	32	21	61	60	25
Mechanisms of injury	Heat-press injury	Contact with heated metal	Heat-press injury	Flame burn	Heat-press injury	Heat-press injury
Follow-up period, month	3	48	12	9	17	30
Injury location	Digit (palmar)	Digits (palmar)	Digits (dorsal)	Digits and hand (dorsal)	Digits and hand (palmar and dorsal)	Digits and hand (palmar and dorsal)
Tajima grade	2	1-3	1-3	1-3	1-3	1-3
Time to flap reconstruction, day	7	2	10	64	1	11, 42
Flap reconstruction	Abdominal flap	Abdominal flap	Reverse radial forearm flap	Reverse radial forearm flap with palmaris longus tendon graft	Abdominal flap	Abdominal flap + free anterolateral thigh flap
Amputation	Yes	Yes	No	No	Yes	No
Secondary reconstruction	None	None	None	None	Toe-to-thumb transfer	Extensor tendon reconstruction

Representative cases are presented for cases 2, 3, and 5.

Case 2

A 32-year-old man sustained a burn injury after his hand became adhered to a 400°C iron object. He presented to our hospital on the day of injury with burn wounds involving the palmar aspects of the right index, middle, and ring fingers (Figure 2A). Surgery was performed two days after injury. After escharectomy, coverage was achieved with an abdominal flap (Figures 2B-2C). After two delayed procedures, flap division was performed one month after surgery. Subsequently, partial flap necrosis and bone exposure developed, necessitating fingertip amputations (Figure 2D). Web space reconstruction was later performed, and the hand remained functional at the four-year follow-up (Figures 2E-2F).

**Figure 2:**
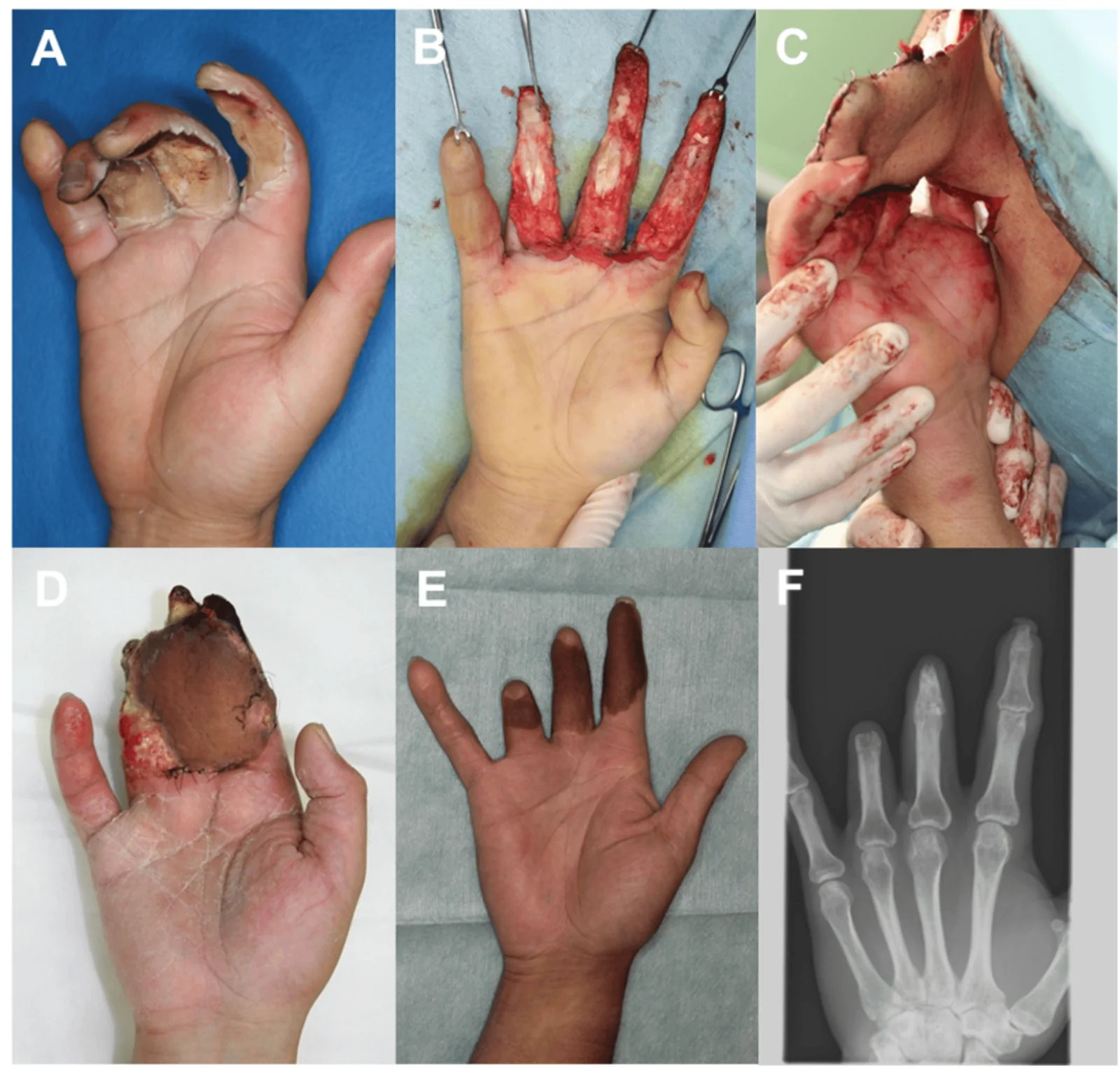
Case 2 A: Photograph at the initial visit showing burn wounds on the palmar aspects of the right index, middle, and ring fingers. B: Intraoperative photograph after escharectomy demonstrating bone exposure. C: Coverage with an abdominal flap. D: Clinical course after flap division showing partial flap necrosis and bone exposure. Fingertip amputations were subsequently performed. E: Clinical photograph at four years postoperatively. F: Radiograph at four years postoperatively.

Case 3

A 21-year-old man sustained a heat-press injury after his hand was caught in a 150°C press machine. He presented to our hospital eight days after injury with burn wounds involving the dorsal aspects of the right index, middle, ring, and little fingers (Figure 3A). Surgery was performed 10 days after injury. After escharectomy, reconstruction was performed with a reverse radial forearm flap (Figures 3B-3E). At one year postoperatively, the patient demonstrated favorable functional outcomes without significant impairment (Figures 3F-3G).

**Figure 3:**
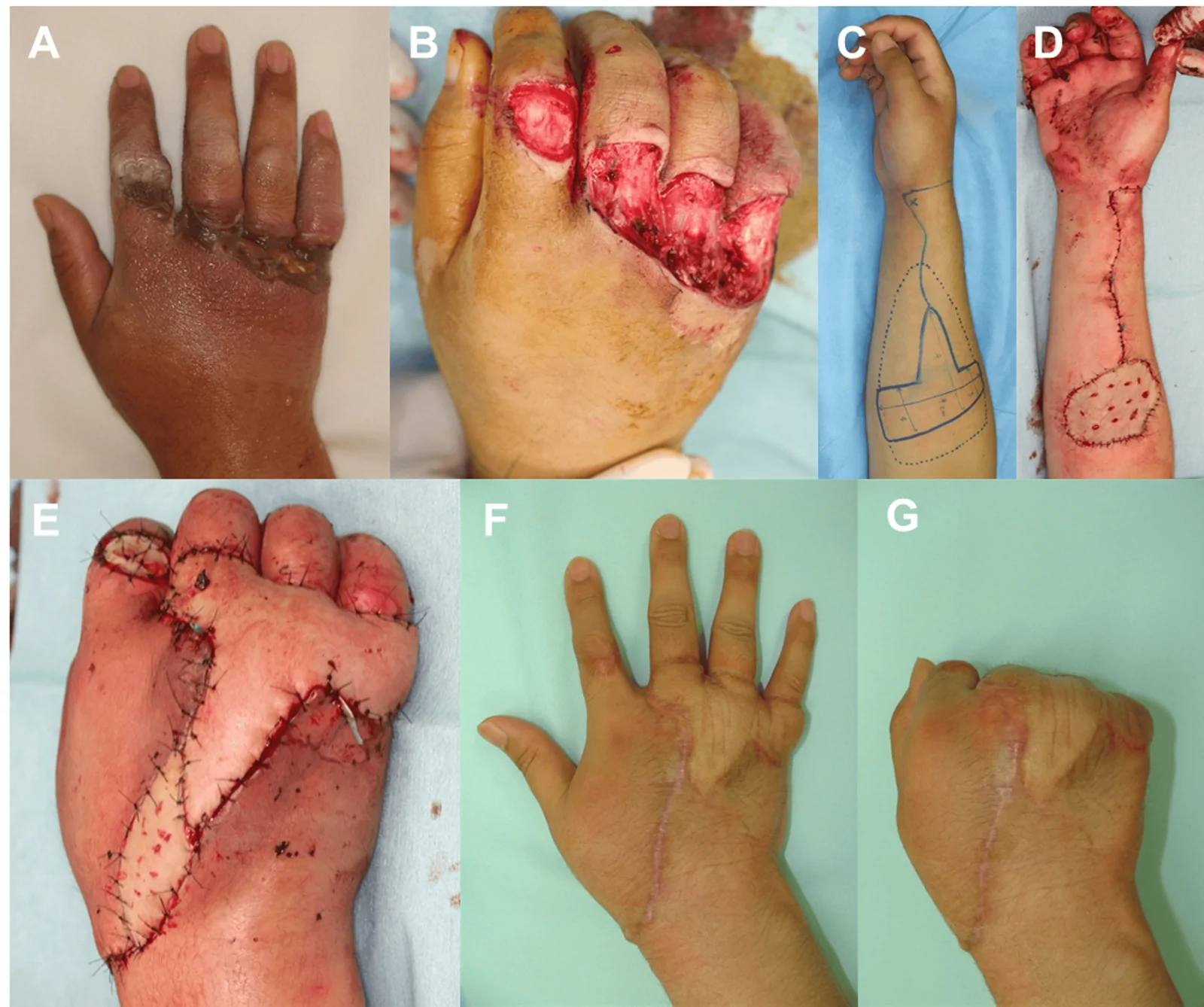
Case 3 A: Photograph at the initial visit demonstrating burn wounds on the dorsal aspects of the right index, middle, ring, and little fingers. B: Intraoperative photograph showing partial necrosis of the extensor tendons. C: Flap design for reconstruction using a reverse radial forearm flap. D: Donor site at the completion of surgery. E: Flap inset at the completion of surgery. F: Photograph at one year postoperatively during finger extension. G: Photograph at one year postoperatively during finger flexion.

Case 5

A 60-year-old woman sustained a heat-press injury after her hand was caught in a high-temperature press machine. She presented on the day of injury with burn wounds involving the palmar and dorsal aspects of the right thumb, index finger, middle finger, and hand (Figures 4A-4B). Debridement and coverage with an abdominal flap were performed one day after injury (Figures 4C-4E). Flap division was performed one month postoperatively, followed by first web space reconstruction three months later. However, osteomyelitis developed in the thumb and index finger (Figure 4F), ultimately requiring amputation at the proximal phalangeal level. One year after injury, toe-to-thumb transfer using a wrap-around flap combined with iliac bone grafting was performed. At one year and five months after injury, the reconstructed hand was being used functionally (Figures 4G-4H).

**Figure 4:**
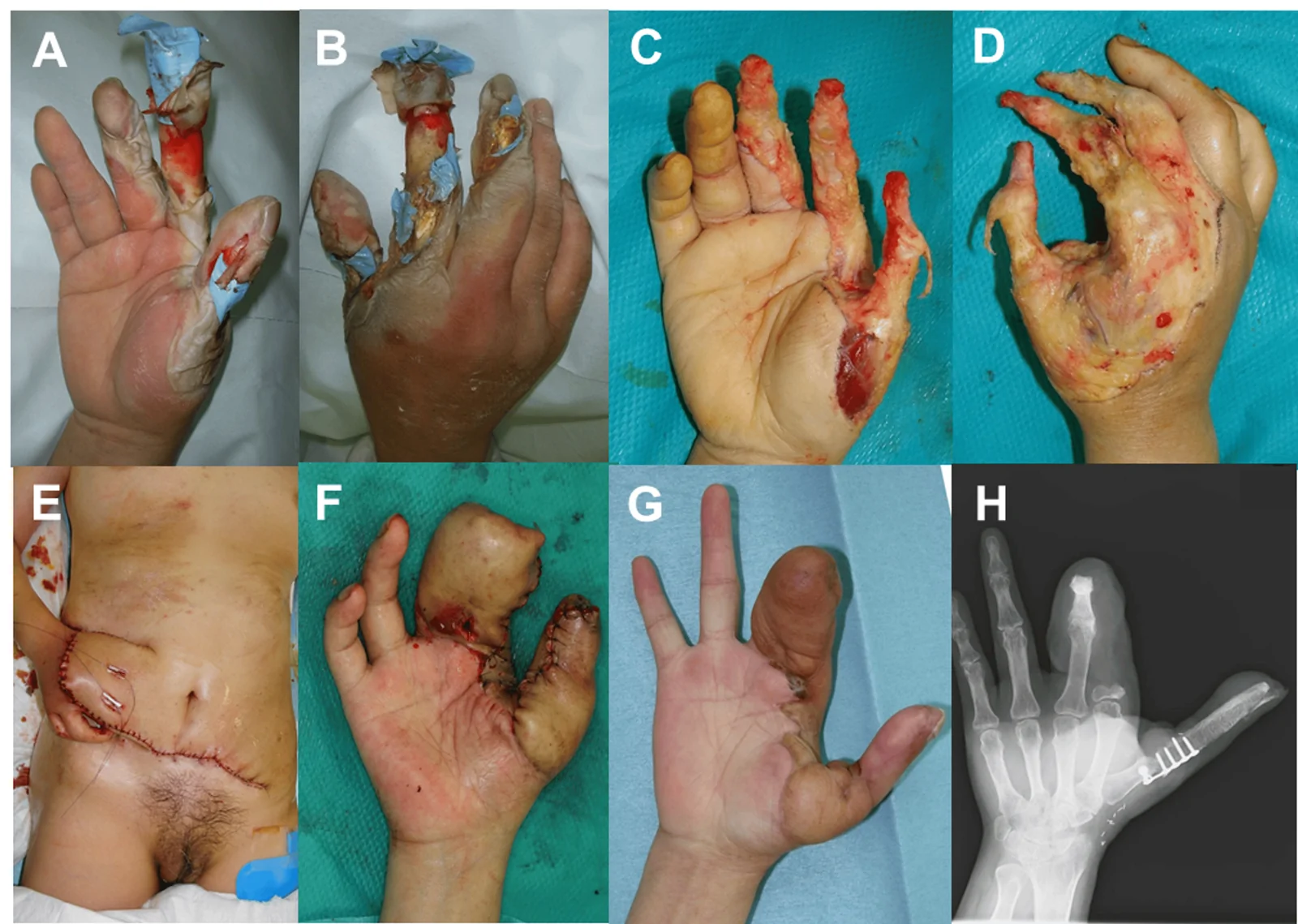
Case 5 A: Photograph at the initial visit showing the palmar aspect of the injury. B: Photograph at the initial visit showing the dorsal aspect of the injury involving the right thumb, index finger, middle finger, and hand. C: Intraoperative photograph of the palmar aspect after debridement. D: Intraoperative photograph of the dorsal aspect after debridement. E: Coverage with an abdominal flap after debridement. F: Photograph after first web space reconstruction showing drainage from the thumb and index finger. Both digits were subsequently amputated. G: Photograph at one year and five months after injury following thumb reconstruction. The patient was able to use the hand functionally. H: Radiograph taken at one year and five months postoperatively.

## Discussion

Hand burns are prone to prolonged treatment courses and frequently result in contracture, deformity, and functional impairment. Therefore, early debridement and early skin grafting are generally considered the standard treatment for hand burns [1]. However, deep hand burns often involve exposure of tendons and bone, and conventional skin grafting alone is insufficient for coverage of poorly vascularized structures. This issue is particularly pronounced in heat-press injuries, in which combined thermal and crush mechanisms lead to progressive tissue damage and make evaluation of tissue viability difficult [2].

Several methods for evaluating tissue viability have been reported, including nuclear medicine imaging [3], angiographic assessment, and laboratory markers reflecting inflammation, muscle injury, or thrombotic tendency [4]. However, none of these methods has become standardized in routine clinical practice. Consequently, intraoperative assessment based on local tissue findings during debridement remains the current standard [2]. Nevertheless, accurate evaluation of tendon and bone viability in the acute phase remains challenging.

The optimal timing of debridement and reconstruction in heat-press injuries remains controversial. Previous studies have reported delayed debridement followed by simultaneous reconstruction approximately one week after injury [2,5], whereas others have advocated staged debridement with delayed reconstruction [6]. By contrast, several authors have recommended early debridement and early flap coverage [7,8]. In five (83.3%) patients, initial debridement was performed one to 11 days after injury, representing an intermediate timing between the early and delayed approaches reported in the literature. Flap coverage was performed simultaneously with the initial debridement in these patients. In the remaining one (16.7%) patient, flap reconstruction was performed in a delayed manner after initial debridement because of the poor general condition associated with extensive burns.

Various reconstructive options for deep hand burns have been described, including distant pedicled flaps such as abdominal flaps, groin flaps, and tensor fascia lata flaps [9]; regional flaps such as reverse radial forearm flaps [5]; and free flaps including free radial forearm flaps [10], latissimus dorsi muscle flaps [11], and anterolateral thigh flaps [5].

In our series, three (50.0%) patients with palmar involvement of the digits ultimately required digital amputation. Direct injury to the digital arteries likely played a major role; however, unlike in degloving injuries, thermal damage to the periosteum itself may also have contributed to progressive necrosis because the periosteal blood supply could not be expected to remain intact.

Although early coverage with abdominal flaps was performed in these patients, delayed necrosis became apparent after flap division, ultimately resulting in amputation. Because the survival of distant pedicled flaps partially depends on blood flow from the recipient bed, maintenance of flap viability may be difficult in deep burns with severe local vascular compromise. Similar concerns regarding the limitations of tissue viability assessment and the indication for distant pedicled flaps have been reported by Minakawa et al. [12].

We also experienced a patient in whom skin grafting was initially selected because tissue viability appeared preserved in the acute phase; however, graft failure subsequently occurred. This finding further highlights the difficulty of evaluating tissue viability in deep hand burns and suggests that the reconstructive strategy selected at the initial operation may significantly influence the final outcome. Conversely, it is possible that early flap coverage with more reliable vascularity might have reduced the need for additional reconstruction.

By contrast, three (50.0%) patients with predominantly dorsal injuries achieved favorable wound healing and functional outcomes. This may be attributable to appropriate debridement, adequate bone management, and successful flap reconstruction combined with tendon reconstruction. These findings suggest that the anatomic pattern of injury may influence prognosis in deep hand burns.

This study has several limitations, including its retrospective design, small sample size, long study period, heterogeneous reconstructive techniques, variable follow-up duration, and limited functional evaluation. Treatment and reconstructive practices may also have changed over the study period. Nevertheless, our findings provide clinically relevant insights into reconstructive strategy selection for deep hand burns.

## Conclusions

Deep hand burns requiring flap reconstruction remain challenging injuries because tissue viability is often difficult to assess in the acute phase. In our series, injuries involving the palmar aspect or both the palmar and the dorsal aspects of the hand were associated with unfavorable outcomes, including delayed necrosis and digital amputation. Our findings suggest that careful assessment of injury depth and anatomic involvement, appropriate selection and timing of flap reconstruction, and close follow-up for delayed necrosis may be important in the management of these injuries.
